# Psychometric evaluation of the exercise-related cognitive errors questionnaire among Chinese emerging adults

**DOI:** 10.3389/fpsyg.2025.1515859

**Published:** 2025-01-28

**Authors:** Mengyao Guo, Jin Kuang, Ting Wang, Fabian Herold, Alyx Taylor, Jonathan Leo Ng, M. Mahbub Hossain, Arthur F. Kramer, Robert Schinke, Zhihui Cheng, Liye Zou

**Affiliations:** ^1^College of Sports Science, Jishou University, Jishou, China; ^2^Body-Brain-Mind Laboratory, School of Psychology, Shenzhen University, Shenzhen, China; ^3^Research Group Degenerative and Chronic Diseases, Movement, Faculty of Health Sciences Brandenburg, University of Potsdam, Potsdam, Germany; ^4^School of Health and Rehabilitation Sciences, Health Sciences University, Bournemouth, United Kingdom; ^5^College of Sport, Health and Engineering, Victoria University, Melbourne, VIC, Australia; ^6^Institute for Health and Sport, Victoria University, Melbourne, VIC, Australia; ^7^Department of Decision and Information Sciences, C.T. Bauer College of Business, University of Houston, Houston, TX, United States; ^8^Department of Health Systems and Population Health Sciences, Tilman J. Fertitta Family College of Medicine, University of Houston, Houston, TX, United States; ^9^Center for Cognitive and Brain Health, Northeastern University, Boston, MA, United States; ^10^Beckman Institute, University of Illinois at Urbana-Champaign, Champaign, IL, United States; ^11^School of Kinesiology and Health Sciences, Laurentian University, Sudbury, ON, Canada

**Keywords:** questionnaire, psychometric evaluation, emerging adults, reliability and validity, six-factor structure

## Abstract

**Background:**

Cognitive errors involve negatively biased or distorted thinking patterns that can hinder effective decision-making. When such a phenomenon occurs in the exercise domain, this is referred to as exercise-related cognitive error. Such exercise-related cognitive errors are typically assessed via a questionnaire, but a validated instrument for the application in Chinese-speaking populations is lacking. Thus, this study aims to validate the Chinese version of the Exercise-related Cognitive Errors Questionnaire (E-CEQ-C) among Chinese emerging adults, a self-report measure to evaluate cognitive errors of context-relevant information related to exercise.

**Methods:**

Following a forward-backward translation of the E-CEQ (*N* = 24 items), the E-CEQ-C and the Chinese version of the Cognitive Distortions Questionnaire (CD-Quest-C) for gathering evidence of criterion-related validity were administered among a sample of Chinese emerging adults (*N* = 376, 29.0% male) through an online survey. After a two-week interval, 105 out of 376 participants attended a re-test of the E-CEQ-C. Item analysis, confirmatory factor analysis (CFA), internal consistency, test–retest reliability, and concurrent validity were analyzed.

**Results:**

The findings from the CFA support the 24-item informed six-factor structure among Chinese emerging adults (χ^2^ = 699.038, RMSEA = 0.073, CFI = 0.919, TLI = 0.904, and SRMR = 0.055). Cronbach’s α of the six dimensions of the E-CEQ-C were all above 0.7. The test–retest reliability coefficients of each subscale and total scale were acceptable, ranging from 0.60 to 0.81. In accordance with the literature, we also observed positive associations between the six dimensions of E-CEQ-C and the constructs of the CD-Quest-C, which provided concurrent validity evidence for the E-CEQ-C.

**Conclusion:**

This study showed that E-CEQ-C is a psychometrically sound measure to assess exercise-related cognitive errors in Chinese-speaking populations.

## Introduction

In recent years, there has been growing evidence that physical activity (PA)—referring to any bodily movement produced by skeletal muscles that requires energy expenditure (Caspersen et al., 1985)—is important for promoting overall health (Blodgett et al., 2023; Hupin et al., 2015; Pearce et al., 2022; Warburton et al., 2006; Yu et al., 2024) and preventing chronic disease (Durstine et al., 2013; Patel et al., 2019). For example, higher levels of regular PA have been shown to benefit a wide range of mental health outcomes including but not limited to improving self-esteem and body image (Huang et al., 2007), enhancing resilience (Zach et al., 2021; Zhang et al., 2022), reducing negative mood (Dinas et al., 2011; Kwan M. et al., 2021; Li et al., 2022; McPhie and Rawana, 2015; Yu Q. et al., 2022), lower levels of depression (McPhie and Rawana, 2015; Kwan M. et al., 2021) improving cognitive functioning (Donnelly et al., 2016; Yu et al., 2021; Zhu et al., 2021), and fostering social connectedness (Bazaco et al., 2016; Deng et al., 2024; Ludyga et al., 2022). Despite a growing recognition of the PA-related health benefits, leading to the World Health Organization’s recommendation that adults aged between 18 and 64 years should engage in at least 150–300 min of moderate-intensity PA or at least 75–150 min of vigorous-intensity PA per week, or an equivalent combination of moderate-to-vigorous PA (Fiona et al., 2020), research suggests that there is a worldwide decline in the overall PA levels across all age groups, particularly among younger adults (López-Valenciano et al., 2021). This is accompanied by an increase in the prevalence of a range of diseases such as obesity (Brittain et al., 2024; Kim et al., 2017; Raiman et al., 2023), diabetes (Schmid et al., 2013; Yuan et al., 2023), and cardiovascular disease (Kazemi et al., 2024; Stelmach et al., 2005), for which a significant risk reduction can be achieved by regular PA engagement (WHO, 2020). Thus, to tackle the public health issue of insufficient levels of PA, there is an urgent need to identify effective strategies to promote PA among the general population, especially among younger adults.

A critical stage for younger adults is emerging adulthood which is defined as a transitional period located between adolescence and adulthood, spanning from about ages 18–29 (Arnett, 2000). Five typical characteristics distinguish this unique period from other life stages, which are (i) identity exploration, (ii) instability, (iii) self-focus, (iv) feeling in-between (adolescence and adulthood), and (v) possibilities (Arnett et al., 2010; Arnett, 2014). This phenomenon has been verified across industrialized countries such as the United States (Arnett, 2007), Australia (Bell and Lee, 2005), and China (Kuang et al., 2023a,b). It is a period for emerging adults to explore various possibilities in love, work, and worldviews, while navigating their way into adult roles (Arnett, 2007, 2014). However, those who fail to adjust to the instabilities in this period are prone to developing mental health problems or adopting risk behaviors (Nelson and Padilla-Walker, 2013; Sussman and Arnett, 2014). Further, emerging adulthood is a critical period in which many healthy lifestyle habits can be formed and continued in the next stage of life (Wood et al., 2017). Comparable to the trends in the general population, several studies have also found that PA levels tend to decline in emerging adulthood (Corder et al., 2019; Deforche et al., 2015; Kwan et al., 2012; Kwan M. Y. et al., 2021). Additionally, many studies reported that a high percentage of college students do not meet the PA recommendations (Quartiroli and Maeda, 2014; Widyastari et al., 2021). According to a recent study in China, 14.4% of college students do not engage in any form of PA, and an additional 41.87% are classified as low-PA participants (Zhao et al., 2022). Although the specific reasons for the high prevalence of physical inactivity are not yet fully clear (Corder et al., 2019; Johnson, 2014), the lack of sufficient amount of PA is associated with many health problems, such as increased risk of cardiovascular diseases (Zhang et al., 2020), diabetes (Carbone et al., 2019), and other chronic diseases (Anderson and Durstine, 2019) in later life. Therefore, investigating patterns of PA and how PA can be promoted in emerging adulthood is critical for disease prevention and health promotion in this population.

In this context, accumulating evidence shows that cognitive errors, typically identified as negatively biased information processing (Lefebvre, 1981), play an important role in hindering people from regular PA engagement (Kgokong and Parker, 2020; Locke and Brawley, 2016, 2017; Lovell et al., 2010), especially in structured and planned forms of PA (Locke and Brawley, 2016) which are known as physical exercise (Caspersen et al., 1985). Previous findings have established associations between cognitive errors and unhealthy thoughts and behaviors, such as dissatisfaction with one’s own physique and weight (Jakatdar et al., 2006), eating disorders (Williamson et al., 1999) and sleeping problems (Alfano et al., 2009). When cognitive errors happen in exercise-related contexts, this is referred to as exercise-related cognitive errors (ECEs). In particular, Gyurcsik and Brawley (2000) suggested that ECEs can lead to lower self-efficacy, lower exercise intentions, which are mirrored in lower exercise attendance.

Based on the cognitive errors model proposed by Drapeau and Perry (2009) and Locke and Brawley (2016) have developed the Exercise-related Cognitive Errors Questionnaire (E-CEQ) which is specifically designed to measure exercise-related cognitive errors. In the original version, 24 initial items representing six kinds of exercise-related cognitive errors, which can be classified into the categories shown in Table 1, were identified.

**Table 1:** Categories of exercise-related cognitive errors and corresponding examples.

Category of cognitive errors	Definition	Example
Catastrophizing	Negative expectations for the future, often believing in the worst outcome without sufficient evidence to support this thought	The last time when you went to the gym, you felt that others were looking in your direction and thought they must be making fun of me because I was doing this exercise incorrectly
Overgeneralization	Generalizing a single negative event to other situations	Because you once failed to exercise for 150 min a week you believe that you will never achieve exercise for that duration in a week
Mental filtering	Focusing only on the negative aspect of an event and ignoring other perspectives which would yield a more comprehensive assessment of the situation	You consider starting an exercise routine, but do not bring this intention into action because you have issues about adhering to routines
Emotional reasoning	Believing that something must be true because one feels that it must be true while ignoring contrary evidence	You plan to exercise today, but do not bring this intention into action because you believe that you will be completely exhausted after the exercise session
The halo effect	The tendency to not engage in a behavior that would foster health (e.g., physical exercise) because one does not engage in a behavior that is detrimental for health (e.g., smoking)	You do not engage in physical exercise because you do not smoke
All-or-nothing	Irrationally concluding that other people’s behavior and external events are related to oneself	A friend asks if you want to start to exercise with him/her. But you do not bring this intention into action because you are relatively skinny

The original version of the E-CEQ was developed within a Western (English-speaking) cultural context (Locke and Brawley, 2016) and has neither been translated into other languages nor validated in non-Western populations. Thus, the purpose of this study is to translate and validate the E-CEQ among Chinese emerging adults. We expect to observe good psychometric properties by analyzing the translated questionnaire factor structure, reliability, and validity. Applying a psychometrically evaluated version of the E-CEQ-C will provide researchers with the opportunity to collect valuable information to determine the intensity and frequency of exercise-related errors among Chinese emerging adults. Collecting such information will add to the development of effective intervention strategies to increase PA participation and adherence among emerging adults with exercise-related cognitive errors.

## Methods

### Participants

A total of 535 participants were recruited through an online self-report survey via the WeChat-based survey program (Questionnaire Star). Out of 535 participants, 376 completed the questionnaires and provided valid responses, yielding a response rate of 70.3%, of which 136 participants were willing to complete a retest after a two-week interval for assessing test–retest reliability. The simple exclusion process was as follows: 78 participants were excluded for failing to provide complete responses to the questionnaires; and 6 participants were excluded for falling outside of the age range from ages 18 to 29. Following the same simple exclusion process, 11 of these 136 respondents’ questionnaires were dropped from the post-test analyses, yielding a response rate of 77.2%. Demographic data are shown in Table 2. Among the 376 participants in the initial test, there were 109 males (29.0%) and 267 females (71.0%), with an age range of 18 to 29 years old (mean age = 21.23 ± 1.96). Among the retest participants, there were 17 males and 88 females, with an age range of 18 to 29 years old (mean age = 21.02 ± 1.71). Informed consents were obtained from all participants included in this study.

**Table 2:** Descriptive statistics of participants for each sample.

	Pre-test sample (*n* = 376)		Re-test sample (*n* = 105)	
	*M*	SD	*M*	SD
Age	21.23	1.96	21.02	1.71
	***N***	**%**	***N***	**%**
**Gender**				
Male	109	29.0	17	16.2
Female	267	71.0	88	83.8
**BMI**				
Underweight	96	25.5	23	21.9
Healthy weight	228	60.6	70	66.7
Overweight	43	11.4	12	11.4
Obesity	7	1.9	0	0
**Employment status**				
Student	326	86.7	95	90.5
Employed or self-employed	38	10.1	8	7.6
Unemployed	12	3.2	2	1.9
**Educational attainment**				
High school or below	4	1.1	0	0
bachelor’s degree	296	78.7	79	75.2
Master’s degree or above	76	20.2	26	24.8

## Measures

### The Chinese version of the exercise-related cognitive errors questionnaire

A total of 24 initial vignettes within the E-CEQ were translated and adapted in the present study after reaching out to the leading authors of the original article for their permission (Locke and Brawley, 2016). This questionnaire consists of six dimensions: Catastrophizing (4 items), All-or-Nothing Thinking (4 items), Overgeneralization (4 items), Mental Filter (4 items), Emotional Reasoning (4 items), and The Halo Effect (4 items). Participants indicated their agreement with these items on a 9-point Likert scale ranging from 1 (“not at all like I would think) to 9 (“exactly like I would think”). The entire process was rigorously conducted following the standards recommended by the International Test Commission guidelines (Hernández et al., 2020). The translation and cultural adaptation of the E-CEQ followed a structured process. First, two psychology undergraduates who are fluent in English independently translated the original E-CEQ into Chinese. A third psychologist then reviewed and resolved discrepancies through expert discussion. Next, a bilingual professor back-translated the Chinese version into English without prior knowledge of the original E-CEQ to ensure consistency. Four experts (one developmental psychologist, one psychometric psychologist, and two health psychologists) reviewed the translated version, resulting in a prefinal version. This prefinal version was tested on 20 undergraduates from an exercise psychology course to assess clarity and comprehensiveness. Feedback from participants was incorporated into the final version after discussion with the researchers and experts, ensuring accuracy and cultural relevance. Efforts were made to minimize differences between the translated version and the original scale as best as possible.

### The Chinese version of the cognitive distortion questionnaire

The Cognitive Distortion Questionnaire (CD-Quest) (de Oliveira et al., 2015), was translated and validated for the Chinese population by Qian et al. (2020) and it was used to assess the frequency and intensity of cognitive distortions or errors. This questionnaire consists of 15 items that cover 15 common types of cognitive errors, such as dichotomous thinking, fortune telling (catastrophizing), emotional reasoning, and selective abstraction. The questionnaire has demonstrated good reliability and validity across emerging adults from Brazil (de Oliveira et al., 2015), the United States (Morrison et al., 2015), Australia (Kostoglou and Pidgeon, 2016), Turkey (Batmaz et al., 2015), and China (Qian et al., 2020). Participants needed to report the frequency and intensity of each cognitive error. For frequency, there were 4 options: (a) never; (b) occasionally (1–2 days), (c) most of the time (3–5 days), and (d) almost always (6–7 days). With respect to the extent to which participants believe a certain cognitive error, there were also four options: (a) not at all, (b) a little (up to 30%), (c) some (30–70%), and (d) very much (over 70%). The scores for each item equaled the sum of the frequency score and the intensity score. Based on the definition of six types of exercise-related cognitive errors in E-CEQ-C, six corresponding items of CD-Quest-C were selected to provide criterion-related evidence. Specifically, this study compared participants’ scores on all-or-nothing thinking of E-CEQ-C to polarized thinking of CD-Quest-C, catastrophizing of E-CEQ to fate judgment of CD-Quest-C, emotional reasoning of E-CEQ-C to emotional reasoning of CD-Quest-C, mental filter of E-CEQ-C to selective abstraction of CD-Quest-C, overgeneralization of E-CEQ-C to overgeneralization of CD-Quest-C, the halo effect of E-CEQ-C to arbitrary inference of CD-Quest-C.

### Statistical analyses

The IBM SPSS Statistics for Windows Version 26.0 was used to perform the following analysis which was supported by previous studies (Wang et al., 2022a; Wang et al., 2022b; Wang et al., 2023; Yang et al., 2023). Mean and standard deviation (SD) were used to describe the score of each dimension of the E-CEQ-C. Skewness and kurtosis were used to validate whether the data were normally distributed. Byrne and Campbell (1999) suggested that absolute values of skewness and kurtosis between 0 and 1.5 indicate normal distribution. Item analysis analysis was used to determine the credibility of each item. It was performed using the critical ratio (CR) method. Among 376 participants, we selected those emerging adults who ranked in the top 27% and the bottom 27% in terms of a total score and each item-related score (David, 1963). Such group differences for each individual item were tested via independent-sample t-tests. If the CR value was less than 3.0, the item was deleted (Hox and Bechger, 1998). Further, item analysis also used item-total correlation analysis to calculate the correlation coefficient between each item and the total score of the questionnaire using the Pearson correlation. If an item had an item-total correlation coefficient lower than 0.30, it was deleted. Moreover, confirmatory factor analysis (CFA) via Mplus 8.0 software was carried out to validate the six-dimensional model of the Chinese version of the E-CEQ. Additionally, internal consistency reliability was evaluated via the total Omega value, which is considered a more robust measure of reliability for multidimensional scales (McDonald, 2013). An Omega value above 0.70 is generally regarded as acceptable for internal consistency, reflecting a high degree of correlation among items. The following fit indices of the model demonstrated an excellent fit: A non-significant chi-square test (*p* > 0.05) indicated a good fit, a root mean square error of approximation (RMSEA) value of ≤0.05 indicated a good fit, a value between 0.05 and 0.08 indicated a reasonable fit, and a value of ≥0.10 indicated a poor fit, a comparative fit index (CFI) value of ≥0.95 indicated a good fit, a value between 0.90 and 0.95 indicated a reasonable fit, and an standardized root mean square residual (SRMR) value of <0.10 indicated an reasonable fit (Kline, 2023). The Cognitive Distortions Questionnaire (CD-Quest-C) was used in the present study as the criterion validity measure for the E-CEQ-C. Criterion-related validity was assessed using Pearson’s correlation coefficient.

## Results

### Distributional properties

Table 3 shows that the skewness and kurtosis of the E-CEQ-C and its subscales all fall between −1.5 and 1.5. These distributional findings have provided evidence for the normal distribution of the data, indicating that the maximum likelihood method could be used for confirmatory factor analysis.

**Table 3:** Mean scores, standard deviations, skewness and kurtosis of E-CEQ-C dimensions.

	*M*	SD	*g*_1_	*g*_2_
Catastrophizing	18.89	6.96	−0.26	−0.75
All-or-nothing thinking	20.85	8.21	−0.22	−0.71
Overgeneralization	14.19	7.04	0.53	−0.22
Mental filter	15.18	8.20	0.47	−0.65
Emotional reasoning	16.98	8.49	0.16	−0.96
The halo effect	11.95	7.35	0.82	0.05
E-CEQ	98.03	38.67	0.22	−0.51

*g1 represents skewness and g2 represents kurtosis.*

### Item analysis

Results are presented in Table 4. All items showed significant differences between the higher scoring group (the top 27%) and lower scoring group (the bottom 27%) (*p* < 0.001), and the cut-off value (CR) ranged from 10.38 to 23.56. The correlation analysis showed that the correlation coefficients between all items and the total score ranged from 0.48 to 0.75, all of which were above 0.30. These data indicated that E-CEQ-C has good item discrimination and good homogeneity for each subscale and the total scale, so there was no need to delete any items.

**Table 4:** The decision values of all items in the E-CEQ and the correlation coefficients between items and the total score of the scale.

Catastrophizing			All-or-nothing thinking			Overgeneralization			Mental filter			Emotional reasoning			The halo effect		
Item	CR	*r*	Item	CR	*r*	Item	CR	*r*	Item	CR	*r*	Item	CR	*r*	Item	CR	*r*
1	10.38^**^	0.48	5	12.92^***^	0.57	9	13.08***	0.57	13	20.88***	0.76	17	24.83^***^	0.75	19	18.35^***^	0.74
2	12.32^***^	0.530	6	14.17^***^	0.60	10	17.16***	0.66	14	18.77***	0.69	18	21.99^***^	0.73	20	15.32^***^	0.65
3	13.11^***^	0.555	7	21.567^**^	0.71	11	11.00***	0.52	15	20.67***	0.77	23	16.87^***^	0.63	21	16.35^***^	0.71
4	12.32^**^	0.504	8	23.56^***^	0.72	12	16.43***	0.68	16	19.83***	0.72	24	18.28^***^	0.70	22	12.48^***^	0.56

^***^ Significance at the 0.001 level (two-tailed).

### Confirmatory factor analysis

**Figure 1:**
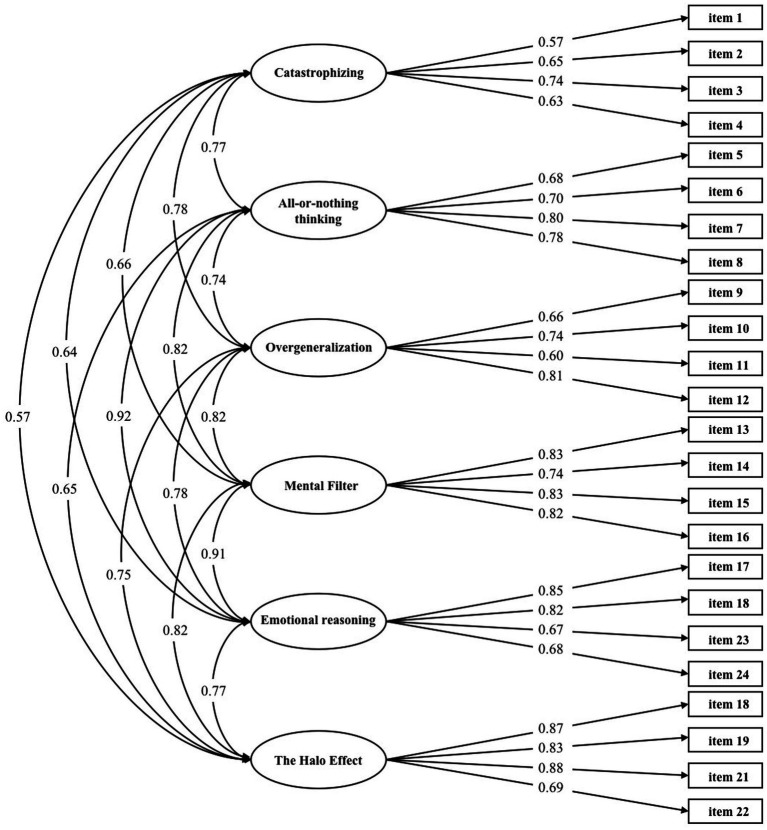
Confirmatory factor analysis: six-factor model of the E-CEQ.

The results of the CFA showed the following model fit indices: χ^2^ = 699.038, RMSEA = 0.073, CFI = 0.919, TLI = 0.904, SRMR = 0.057, GFI = 0.855, AGFI = 0.814, NFI = 0.884, and IFI = 0.920. CFA was conducted on the 24-item scale, as shown in Figure 1. All correlations between the single factors were statistically significant. The total Omega value was 0.88 in this analysis.

### Criterion validity

Results (Table 5) indicated that the correlation coefficient between exercise-related cognitive errors and cognitive distortions was 0.333 (*p* < 0.01). Notably, fate judgment of the CD-Quest-C was positively correlated with catastrophizing of the E-CEQ-C; polarized thinking of the CD-Quest-C was positively correlated with all-or-nothing thinking; overgeneralization of the CD-Quest-C was positively correlated with overgeneralization of the E-CEQ-C; selective abstraction of the CD-Quest-C was positively correlated with mental filter of the E-CEQ-C; emotional reasoning of the CD-Quest-C was positively correlated with emotional reasoning of the E-CEQ-C, and arbitrary inference of the CD-Quest-C was positively correlated with the halo effect of the E-CEQ-C, with correlation coefficients ranging from 0.136 to 0.293 (*p* < 0.05).

**Table 5:** Correlation analysis between the Exercise Cognitive Errors Questionnaire and its factors with the criterion measure.

	Fate judgment	Polarized thinking	Overgeneralization	Selective abstraction	Emotional reasoning	Arbitrary inference	Cognitive distortions
Catastrophizing	0.29^**^	0.27^**^	0.26^**^	0.24^**^	0.15^**^	0.26^**^	0.35^**^
All-or-nothing thinking	0.15^**^	0.14^**^	0.18^**^	0.16^**^	0.09^**^	0.14^**^	0.21^**^
Overgeneralization	0.28^**^	0.22^**^	0.27^**^	0.25^**^	0.17^**^	0.16^**^	0.35^**^
Mental filter	0.21^**^	0.22^**^	0.23^**^	0.18^*^	0.12^**^	0.20^**^	0.28^**^
Emotional reasoning	0.19^**^	0.22^**^	0.24^**^	0.24^**^	0.14^**^	0.18^**^	0.29^**^
The halo effect	0.08^**^	0.12^**^	0.20^**^	0.19^**^	0.14^**^	0.18^**^	0.22^**^
Exercise cognitive errors	0.24^**^	0.23^**^	0.27^**^	0.25^**^	0.16^**^	0.24^**^	0.33^**^

*Significance at the 0.05 level (two-tailed), correlation is significant; ** Significance at the 0.01 level (two-tailed), correlation is significant.

### Test–retest reliability

The results of test–retest reliability analysis for the questionnaire indicated that correlation coefficients between the pre-test and the re-test of all subscales and total scale were significant (*p* < 0.01). The overall test–retest reliability coefficient for the total questionnaire was 0.81. The test–retest reliability coefficients for catastrophizing, all-or-nothing thinking, overgeneralization, mental filtering, emotional reasoning, and the halo effect were 0.60, 0.74, 0.63, 0.76, 0.70, and 0.67, respectively.

## Discussion

In the present study, the psychometric characteristics of the Chinese version of the Exercise-related Cognitive Errors Questionnaire were examined among Chinese emerging adults (*N* = 376). Results of the CFA demonstrated that the 24-item, six-dimensional model displayed a good model fit with highly loaded items. Additionally, the E-CEQ-C was significantly associated with constructs of the CD-Quest-C, providing concurrent validity evidence for this adapted version in Chinese culture. Further, the total McDonald’s Omega of the E-CEQ-C was 0.88, indicating sufficient reliability. The test–retest reliability coefficients of the six dimensions were also all in the acceptable range (from 0.60 to 0.81). Collectively, the Chinese version of the E-CEQ is a reliable instrument to evaluate the degree of exercise-related cognitive errors with acceptable levels of psychometric properties.

We found that our results of CFA supported the 24-item, six-dimension structure of the E-CEQ-C, which slightly different from the original 16-item, three-dimension structure of the E-CEQ. A possible explanation for this inconsistency is that six kinds of cognitive errors described by Drapeau and Perry (2009) also exist in the exercise-relevant context in the Chinese emerging adult population. There is also convincing evidence to verify the existence of six categories of exercise-related cognitive errors among Chinese emerging adults. Firstly, the results of the item analysis verified the credibility of every item. Second, the results from the CFA support the 24-item, six-factor structure among Chinese emerging adults (χ^2^ = 699.038, RMSEA = 0.073, CFI = 0.919, TLI = 0.904, and SRMR = 0.055), with factor loadings of each item in a satisfying range from 0.57 to 0.88. Additionally, McDonald’s Omega values document that the E-CEQ-C and each of the six subscales are internally reliable and form a coherent set of the construct. Lastly, the test–retest reliability ensures the consistency and reproducibility of results obtained from the E-CEQ-C over time.

In the previous study, Locke and Brawley (2016) observed a moderate-sized relationship between the E-CEQ and the general CEQ developed by Lefebvre (1981). In accordance with the literature, we also observed positive associations between subscales of the E-CEQ-C and constructs of the CD-Quest-C in the sample of Chinese emerging adults (as shown in Table 5). Moreover, some subscales of the E-CEQ-C (i.e., all-or-nothing thinking, overgeneralization, mental filter, emotional reasoning, the halo effect) not only had significant relationships with corresponding subscales of the CD-Quest-C (i.e., polarized thinking, overgeneralization, selective abstraction, emotional reasoning, arbitrary inference) but also have stronger associations with other subscales (i.e., overgeneralization, fate judgment or selective abstraction, overgeneralization). This result may reflect the similarity between negative thinking resulting from cognitive errors from the same cluster (Drapeau and Perry, 2009).

There are several implications of this validation study for advancing exercise-related behaviors and decision-making in Chinese emerging adults. Contextually appropriate measurement tools can be helpful to assess existing cognitive challenges that may impact how individuals adhere to healthier exercise practices. Notably, cognitive errors can result in a decreased motivation for exercise, elevated stress and possible self-criticism, exercise-related self-efficacy, and adverse mental and physical health outcomes (Locke and Brawley, 2017; Locke and Brawley, 2018). Tackling these challenges would require precision approaches in measuring the problems and evaluating the effectiveness of adopted interventions. This validated instrument can play critical role in supporting appropriate measurement and improving exercise behaviors in Chinese emerging adults. However, the scope of how this scale and examined constructs are relevant in the context of current and evolving social and cultural contexts in China can be future research agendas alongside examining the role of evidence-informed practices to improve exercise an associated health outcomes.

## Limitations

The present study has some strengths and limitations. In terms of strengths, on the one hand, we followed rigorous standards of forward-backward translation (Beaton et al., 2000) to translate the E-CEQ into Chinese. On the other hand, we carried out a complete cross-cultural adaptation of the questionnaire. Factor structure and criterion validity of the E-CEQ were tested, and its internal consistency and test–retest reliability were validated. However, there are also some limitations. Firstly, this study was restricted by many factors as the data was collected through convenience sampling, which means the sample may be vulnerable to selection bias and lacks clear generalizability. Second, the self-report measures adopted in this study could not rule out the possibility of social desirability bias. Besides, this study was carried out among emerging adults, which indicates that the applicability of the E-CEQ still needs to be tested among other populations (e.g., older adults, adolescents). Therefore, we recommend that based on this study, future research should prioritize addressing the following research gaps. First, future studies should be carried out in clinical populations, such as patients with mental disorders to expand the applicability of the scale in the Chinese population. Second, future research should seek to compare the differences in the magnitude of exercise-related cognitive errors among different groups, such as different ages, genders, socioeconomic status, and countries, to conduct cross-sectional comparisons. Third, it is of high practical relevance to examine in future studies the ecological validity of the E-CEQ-C by investigating the relationship between the magnitude of individuals’ exercise-related errors and their PA levels assessed by self-report (e.g., via validated physical activity questionnaires) or device-based assessment method (e.g., via accelerometer). Moreover, future studies should seek to elucidate the neurophysiological basis for exercise-related cognitive errors using various imaging techniques such as electroencephalography (Yu C. et al., 2022), functional near-infrared spectroscopy (Ekkekakis, 2009) and eye-based measures (Zou et al., 2023).

## Conclusion

In the current study, we translated and evaluated the psychometric properties of the Exercise-related Cognitive Errors Questionnaire among Chinese emerging adults. Our analyses confirmed that the E-CEQ-C is a valid and reliable instrument for assessing exercise-related cognitive errors in this population. Based on our findings, the E-CEQ-C can be utilized to broaden our knowledge of the psychological mechanisms underlying Chinese emerging adults’ PA behaviors. Such a deeper understanding of the psychological mechanisms will help to better inform the development of more effective intervention approaches and provide actionable guidance to promote exercise participation and adherence among emerging adults with exercise-related cognitive errors.

## Data Availability

The datasets presented in this study can be found in online repositories. The names of the repository/repositories and accession number(s) can be found at: https://doi.org/10.31219/osf.io/gydpx.
